# A Developer-Oriented Framework for Assessing Power Consumption in Mobile Applications: Android Energy Smells Case Study

**DOI:** 10.3390/s24196469

**Published:** 2024-10-07

**Authors:** Claudiu Groza, Apostol Dumitru-Cristian, Marius Marcu, Razvan Bogdan

**Affiliations:** Faculty of Automation and Computers, Polyethnic University of Timisoara, 300006 Timisoara, Romania; claudiu.groza@cs.upt.ro (C.G.); dumitru.apostol@student.upt.ro (A.D.-C.); marius.marcu@cs.upt.ro (M.M.)

**Keywords:** power consumption, software design patterns, bad patterns, energy code smells

## Abstract

Currently, people spend a lot of time using their mobile devices. With such ubiquity of mobile devices in our daily life, battery capacity and quality are of utmost importance. Running software applications (called apps) are one of the major factors influencing the power consumption in mobile devices. In order to meet user needs, mobile apps are becoming inherently complex and resource greedy. Therefore, fulfilling these requirements at the pace imposed by the market may degrade software construction quality and insert so-called energy code smells: bad patterns in the source code of an app that indicate a deeper problem and adversely affect power consumption. This work proposes a developer-oriented framework for identifying and fixing patterns via analyzing different application code flavors in a user-driven test scenario. A special app was designed in order to validate the Android implementation of the proposed methodology. The study results have shown significant improvement regarding energy efficiency after correcting one or more energy code smells, with a 4 to 30 percent decrease in battery drain. Additionally, the power consumption signature term is defined in the context of mobile applications. This paper presents a developer-oriented framework for assessing power consumption in mobile applications. Our key contributions include identifying significant energy code smells, demonstrating their impact on power consumption, and providing a toolset for developers to improve energy efficiency.

## 1. Introduction

In 2024, the number of smartphone users worldwide is 4.88 billion, meaning 60.42% of the world’s population owns a smartphone. Some users have more than one smartphone, which totals around 7.21 billion active smartphones in the world currently [1]. With such ubiquity of mobile devices in our daily life, battery capacity and application quality are of utmost importance. A mobile application software (mobile app) is an application software that runs on mobile systems like smartphones, tablets, or wearables. These devices are powered from batteries which are usually with an average battery capacity of around 3000–4000 mAh in modern smartphones. This implies that battery capacity is severely constrained by the consumer device size characteristics. Hence, if the size of a battery cannot be substantially increased, then an optimal management of power consumption on these devices is critical. Mobile applications require efficient processing power management to optimize battery life without sacrificing performance.

Moore’s Law, once predicting a continuous exponential increase in processing power, has now reached a plateau. This shift emphasizes the growing importance of optimizing software, rather than relying on hardware improvements alone. Our framework directly addresses this challenge via focusing on the detection and correction of energy code smells, which significantly affect power consumption in modern mobile applications. Unfortunately, there is no known rule related to batteries because ions, which transfer charge in batteries, are large, and they take up space, as do anodes, cathodes, and electrolytes [2]. Microprocessors’ processing power is limited by lithography technology development, but significant improvements in battery capacity can only be achieved by changing the intrinsic chemistry [2]. For instance, most modern smartphones are equipped with batteries of 3000 to 4000 mAh capacity, which imposes strict constraints on energy availability. Our framework is designed to help developers optimize power consumption under these hardware limitations, ensuring that mobile applications remain responsive and efficient while using minimal energy.

According to the TIME Mobility Poll, the most popular and desired improvement for a mobile device is the battery capacity, bringing attention to the end-user perspective. Other improvements like lower price, larger or smaller screen size, better voice recognition capabilities, or sturdier device have been overcome by a longer battery life [2]. Considering the above limitations, a software construction workflow which enables power consumption tracking can help application developers to deliver energy-efficient applications.

One of the major factors influencing the power consumption in mobile devices is the software applications that run in such environments. They require increased processing power, thereby raising the capacity and lifetime expectations of a battery. Mobile applications are becoming complex software systems that must continuously adapt and fit a large set of user requirements [3]. Thus, fulfilling these requirements may degrade software quality and explicitly insert new smells in the source code.

While this study focuses on the Android platform, which could limit the generalization of our findings, the fundamental principles behind energy code smells and their correction are applicable across mobile operating systems. Future research should test the framework on other platforms, reinforcing the importance of energy-efficient software development in the face of hardware limitations. A code smell or a bad smell is a pattern in the source code of a program that indicates a deeper problem or bad design practices. Code smells which adversely affect the power consumption of mobile apps can be called energy code smells [4]. The process of correcting a code smell is called refactorization. The decision of not refactoring code affected by bad smells will not result in the application stopping running but it may slow down development or increase the risk of bugs and system malfunctioning in the future. Deferring the action of refactorization may contribute to the technical debt of the system. Therefore, refactoring may improve the maintenance phase of an application. The identification of code smells can be performed using static analysis (code analysis) because they are exclusively related to code patterns. Thus, a static analysis tool does not need to run the application, as opposed to behavior patterns tools which need application runtime information. Adopting the activity of refactorization as a part of the software construction process will inherently improve the maintainability of an application.

Developing a methodology that helps developers to track and fix energy code smells should contain the following base requirements:

- Energy code smell detection and refactorization;
- Power consumption signature before and after refactorization;
- Battery drain measurement in user-driven context;
- Results’ analysis: regression or improvement.

In order to achieve an increased adoption of integrated quality tools, the implemented solutions of the above requirements must be automated, extensible, and reusable [5].

The following research questions will be addressed using the work presented in this paper:

- RQ1: Which are the recommended battery characteristics to be analyzed in order to spot power consumption changes?
- RQ2: Is there a quantifiable correlation between energy code smells and power consumption for mobile applications?
- RQ3: Can the software power consumption signature be used to assess the quality of different versions of an application?

This paper contributes to the growing need for energy-efficient software via (1) identifying critical energy code smells that degrade power performance, (2) proposing a robust developer-oriented framework designed to detect and refactor these inefficiencies, and (3) validating the framework’s effectiveness through significant improvements in battery life, underscoring its practical value for developers aiming to create energy-conscious mobile apps.

## 2. Related Work

Understanding the intricate relationship between code quality and energy efficiency in mobile application development has become a focal point of research. Several studies have laid significant groundwork in this domain, establishing a taxonomy for identifying and categorizing design flaws that escalate energy consumption in Android apps. Notably, Carette et al. [6] demonstrated that correcting code smells like Internal Getter/Setter, Member Ignoring Method, and HashMap Usage can significantly reduce a mobile application’s energy consumption. Their methodology, however, involves an external monitor (Yocto-Amp), reducing its usability and adoption by mobile developers.

Mario Linares-Vásquez et al. [7] designed a measurement framework focused on accuracy using an external power monitor device. While accurate, this approach is not easily integrable into common projects by mobile developer teams. Similarly, Ricardo Prez-Castillo and Mario Piattini [8] explored the effects of the god class anti-pattern on power consumption, concluding that a more maintainable architecture might negatively impact energy consumption. However, their study was limited to traditional Java applications, not Android apps.

Further, Ching Kin Keong et al. [9] established a direct link between software quality metrics and power consumption, showing strong correlations with metrics like McCabe cyclomatic complexity, number of parameters, and method lines of code. While offering hints on power consumption related to software quality metrics, Litke et al. [10] and subsequent studies [11] provided insights into design patterns and good coding practices, without specifically addressing energy code smells.

Ding Li and William G. J. Halfond [12] investigated common best practices for energy saving, providing general guidelines for developers. They also examined power consumption at the source-line level [13,14], identifying correlations between application states and power consumption. Sona Mundogy and Sudarshan K [15] investigated Google best practices to improve performance and power consumption via refactoring different code segments.

Class Wike et al. [16] compared energy consumption levels across mobile applications delivering the same services, proposing a methodology to profile different apps using user-driven test cases. Marius Marcu and Cosmin Cernazanu [17,18] highlighted that the power signature is device-specific, influenced by usage patterns, though their findings cannot be generalized across different mobile applications.

Hecht et al. [19] examined the impact of fixing Android code smells on user interface and memory performance, without addressing power consumption. However, the correction of code smells improved Dalvik machine metrics, contributing to performance concerns. Additionally, comprehensive surveys like [20] target multiple performance characteristics, including energy consumption, for Android applications.

Expanding on these foundational studies, a research endeavor created a dataset of 60 Android apps for empirical analyses, utilizing tools like aDoctor, dmtracedump, BatteryStats, and Systrace to examine the energy implications of code smells. The findings resonated with previous research on the adverse effects of specific energy smells like Internal Setter, Leaking Thread, and Slow Loop. Moreover, the study unveiled co-occurrence patterns of code smells, offering potential avenues for optimization and targeted refactoring endeavors.

Other studies, like the one using JDeodorant for code smell detection and refactoring in Android apps from F-Droid [9], showed significant energy impact in two out of three apps but found no clear correlation between specific code smell permutations and energy consumption. Evaluations of tools like EcoAndroid and ecoCode have also been integral in advancing automatic refactoring techniques for energy efficiency in Android apps.

The relationship between code smells and power consumption has been further explored through manual refactoring techniques, with tools like Microsoft Joulemeter estimating energy consumption. These studies generally observed significant reductions in power consumption post refactoring. Moreover, sociotechnical aspects, such as community dynamics, have been shown to influence code quality and maintenance decisions significantly, as revealed through an analysis of major open-source project releases.

In summary, integrating energy considerations into code maintenance practices is crucial. Investing in tools and methodologies for detecting and alleviating code smells can significantly improve the energy efficiency of mobile applications. These studies underscore the importance of multifaceted approaches, combining technical, empirical, and community-aware strategies to enhance code quality and sustainability in software engineering.

## 3. Theoretical Background

### 3.1. Energy Code Smells

Energy code smells are defined as “implementation choices at source code level (code patterns) that make a suboptimal usage of the hardware resources underneath” [4]. As a result, these patterns increase the overall power consumption of the device. Energy code smells are closely tied to the programming language and platform they operate on. In Androids, the most studied energy smells are HashMap Usage (HMU), Internal Getter/Setter (IGS), and Member Ignoring Method (MIM) [6,19,20,21].

We chose HMU, IGS, and MIM energy code smells because they are the most studied and have shown significant impacts on energy consumption in prior research [1,2].

**HashMap Usage.** In Androids, memory usage patterns significantly impact application performance. Continuous memory allocation and deallocation, along with intensive garbage collection calls, can degrade performance. Android best practices suggest using alternatives like ArrayMap, SimpleArrayMap, and SparseArray instead of HashMap. An example of HashMap Usage is provided in Listing 1.

**Listing 1.** HashMap usage smell.HashMap<Integer, Object> map; void append(int id, Object object) {   map.put(id, object); }

**Internal Getter/Setter.** IGS occurs when a field is accessed through a getter/setter within its declaring class. This indirect field access can affect application performance. While using getters and setters is considered good practice in languages like C++, C#, and Java due to compiler inlining, in Androids, virtual method calls are more expensive than instance field lookups [20,22]. Correcting this smell involves directly accessing the field within the declaring class. Listing 2 provides a trivial example of IGS.

**Listing 2.** Internal Getter/Setter smell.String firstName; String lastName; public String getFirstName() {   return firstName; } public String getLastName() {   return lastName; } public String getFullName() {   return getFirstName() + "," + getLastName(); }

**Member Ignoring Method.** A method that does not need to access an object’s fields or is not a constructor should be made static. Static methods make invocation processes 15–20% faster and enhance readability via ensuring that the method does not alter the object state [20,22]. An example of MIM smell is presented in Listing 3.

**Listing 3.** Member Ignoring Method smell.private getTimeDifference(Calendar pastDate) {   Calendar today = Calendar.getInstance();   int difference = today.getTimeInMillis()          - pastDate.getTimeInMillis();   return difference; }

### 3.2. Energy Measurement

In the context of mobile devices, the battery fuel gauging subsystem estimates the remaining energy in the battery. There are two primary methods for monitoring battery capacity: voltage measurements and current measurements [21,23]. 

**Figure 1:**
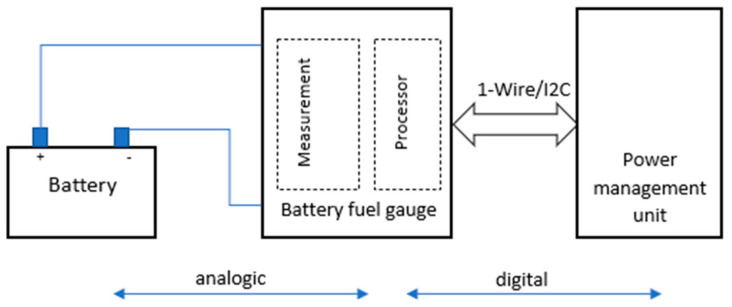
Block diagram of a battery monitoring system.

Figure 1 illustrates a generic battery measurement and management system. It includes a battery fuel gauge for measuring and estimating battery parameters like state of charge, state of health, and available energy, and a power management unit that collects status data from the battery to send notifications or high-level data to the operating system or applications. The analog part of the design measures battery current, voltage, and temperature, which are necessary for accurate battery parameter estimation [22,24]. These measurements are sent to a microprocessor unit in the digital part, which implements the fuel gauging algorithm.

The most basic algorithm for estimating battery capacity involves measuring battery voltage and referencing a lookup table. The lookup values are based on the voltage curve of a Lithium-Ion battery as a function of charge state. A more precise solution is the Coulomb counting technique, which calculates the state of charge via measuring battery current and integrating it over time. However, this method suffers from long-term drift and lack of a reference point. Most algorithms combine voltage measurement with current flow integration to improve estimation accuracy.

### 3.3. Power and Energy Signatures

Application power signature encompasses the attributes related to the notion of power consumption behavior in the context of applications. The distinction between different consumption behaviors gives developers a clearer understanding of delivered application services [23].

Quantitative and automatic measures of power traces open up a new area of research. Therefore, we can dare to introduce the following terms in the context of mobile application development:

- Application energy class or label;
- Power consumption regression testing;
- Energy efficient oriented development.

## 4. Experimental Setup

### 4.1. Measurement Methodology Definition

This chapter describes the primary components of a research framework for gathering power consumption data for mobile applications. The concepts are presented at a high abstraction level, making them reusable across different mobile application systems. An Android implementation of this methodology will be introduced in the next chapter.

Our methodology involves measuring average current, instantaneous current, charge counter, and energy counter to analyze power consumption patterns.

**Figure 2:**
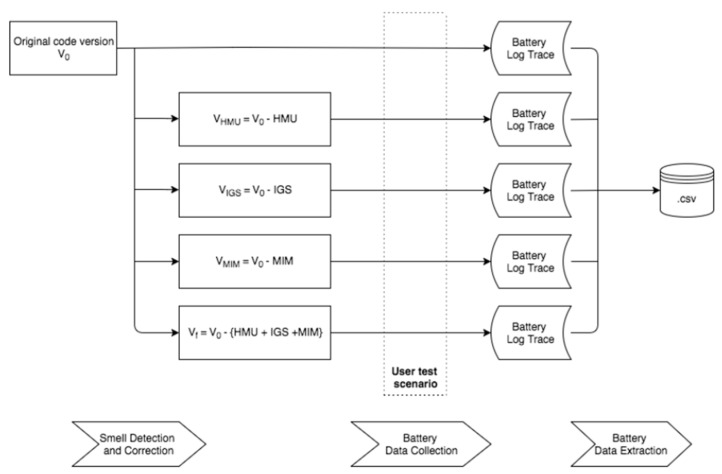
The workflow of the proposed methodology.

The basic steps are distinguished as follows:

- Initial code base definition;
- Detection and correction of targeted code smells and application flavors;
- Battery measures collection during application runs;
- Measurements data extraction and processing;
- Overview of the proposed solution, as is shown in Figure 2.

**Smell Detection and Correction**. This step encapsulates the process of producing different application flavors based on a catalog of code smells to be corrected. The input–output relation of the entities involved in this step is defined in Algorithm 1.
**Algorithm 1:** Smell Correction and Flavor Generationabstract function PurgeSmells (*baseApp, smell Set*); **Input:** Initial code version baseApp, the catalog of code smells to be corrected smell Set **Output:** A appFlavor for each code smell contained in the catalog and base App flavor

**Data Collection**. The data collection step records the power consumption of a user-driven scenario run as power traces. A power trace is a timestamped power measurement collected during the execution of a program (application) [7]. A user-driven scenario is composed of a set of user activities that cover several system components, focusing on how a subject uses the system in different roles [8]. The entities involved in this step are presented in Algorithm 2.
**Algorithm 2.** Power traces collection.abstract function RunAndMeasure (*appFlavors, scenario*); **Input:** The set of appFlavors to be executed given the user-driven scenario **Output:** Timestamped powerTraces for each flavor contained in appFlavors

**Data Extraction**. This phase consists of two independent substeps. The first defines the connection and extraction of power traces, specific to the platform on which the scenario is executed. The second step involves power trace processing and exporting to a universal format. A universal output format provides the opportunity to easily integrate with other developer tools. The program flow, which transforms raw data into a .csv file, is defined in Algorithm 3.
**Algorithm 3.** Power traces extraction.abstract function Extract (*powerTraces*); **Input:** The powerTraces data on which the extraction to be performed
**Output:** An universal format csv File

### 4.2. Applications under Analysis

The experiment presents a crafted Android application containing a catalog of three artificially created energy smells: HMU, IGS, and MIM. The subject application can be investigated and accessed via a public repository [9]. The current implementation ensures each code smell can be fixed and studied individually, providing a flexible platform for creating high-fidelity user scenario tests. The user interface simulates a common mobile application with different screens and a navigation bar.

The subjects of the investigation are the three Android code smells previously described. Table 1 presents the correspondence between code versions and corrected code smells. The initial flavor V0 of the app was used to define one or more user test scenarios. Tests were run on all derived flavors (VHMU, VIGS, VMIM, Vf) of the app to ensure initial functionalities remain intact after the code smell correction step.

### 4.3. Measurement Methodology

**Physical environment**. The mobile device used for the experiment was a Motorola Nexus 6 (Chicago, IL, USA), running stock Android 7.0 (Nougat). This environment was chosen due to permission changes in newer Android versions. The instrumentation test ran through an ADB Wi-Fi connection to keep the device disconnected from a USB power source.

**Minimization of external factors**. Repeatability and reproducibility of the experiment were achieved by respecting the physical environment and following specific rules. The Wi-Fi router used for the ADB connection needed to provide optimum signal strength and limit network peers to the machine orchestrating the tests and the mobile device running the tests. The router had no external Internet connectivity, ensuring consistent network parameters without influencing power consumption between runs.

Each experiment scenario started from a common set of battery parameters. The battery needed to be fully charged (100% capacity) to minimize the implications of internal battery chemistry.

**User test scenario.** The user test scenario was specified before any code correction. Execution time was inspired by the average amount of time a user spends daily within an application, ranging from 1 h to 1 min per day [10]. The current configuration set a scenario lasting about 15 min, easily adaptable according to the application under measurement. The recorded test scenario included common user interactions like tab navigation, button pressing, and short-term computations.

**Code smell detection and correction**. The Lint static code analysis tool confirmed the presence of artificially created code smells. Corrections were applied manually, and the detection step was repeated to ensure problems were fixed.

**Power consumption trace**. A power consumption trace was collected before and after a user executed a specific action associated with a button.

**Measurement parameters**. The independent variables of the experiment were the flavors derived after the code smell correction step and initial version. The dependent variables were represented by the battery characteristics.

Device power consumption can be determined for Android devices which include a battery fuel gauging system. The fuel gauging chip integrated on each device may differ and the measured characteristics as well. Currently, the Android battery API supports the following battery fuel gauging properties found in Table 2 [24,25,26,27,28].

To further the applicability and validation of our framework, the replication package is presented in [29]. This will enable other developers and researchers to verify the impact of energy code smells on power consumption, ensuring that our solution can be effectively used to optimize energy efficiency in a wide range of mobile apps.

## 5. Experimental Results

### 5.1. Data Analysis

This chapter provides an analysis of the constituent raw data generated using the power consumption traces obtained in previous chapter. The next few lines define a set of formulas and operators with the purpose of quantifying differences between different data traces. The application under analysis is a widely used mobile app with high user engagement, providing a robust context for studying energy code smells. In the new Android versions, the data analysis is performed with the help of the adb Systrace.

An important parameter that may give an immediate understanding over the improvement/regression of two distinct application versions is the difference between start and final values of a battery characteristic:

$$\Delta_{C}=C_{I}-C_{F}$$

where *Ci* is the initial value of C characteristic and *Cf* is the final value of C characteristic.

The arithmetic average operator applied to a characteristic may provide insights regarding average power consumption over a period of time:

$$\left(\;C_{a\;v\;g}=\frac{1}{n}\;\sum_{i=1}^{n}XC_{i}=\frac{1}{n}\left(C_{1}+C_{2}+\cdots +C_{n}\right)\;\right)$$

where *n* is the number of traces and *Ci* is the measured value at the *i*th step.

The median operator is used to find an indication towards the central tendency of a battery characteristic:

$$\left(\;C_{m\;e\;d}=median\left\{C_{1},\;C_{2},\;\cdots ,\;C_{n}\right\}\;\right)$$

where *n* is the number of traces and *Ci* is the measured value at *i*th step.

For calculating the percentage difference between two values, the formula is introduced is as follows:

$$\left(D_{C}\left(\%\right)=\frac{C_{b}-C_{a}}{C_{a}}\times 100\right)$$

where *Cb* is the new value and *Ca* is the original value.

### 5.2. Battery Capacity

**Figure 3:**
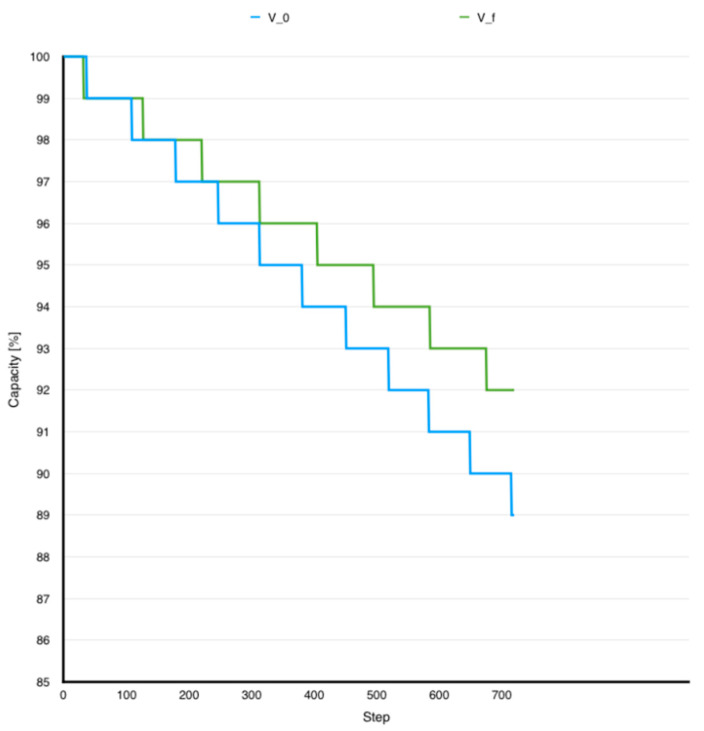
Capacity unfolding V_0_ vs. V_f_.

Figure 3 presents the battery capacity characteristic evolution in two conditions: initial application with all the smells inside and the same application with all the smells solved using the correct solutions. Table 3 shows a battery capacity comparison between examined code flavors in battery capacity reported to V_0_.

The following observations were made based on previously analyzed data:

- All corrected versions were more energy efficient than the initial version.
- The differences between final capacities could be observed in terms of battery percentage shown in mobile device status bar.
- HMU code smell contributed the most to battery drainage.
- IGS code smell had the smallest contribution in overall consumption.

### 5.3. Charge Counter

**Figure 4:**
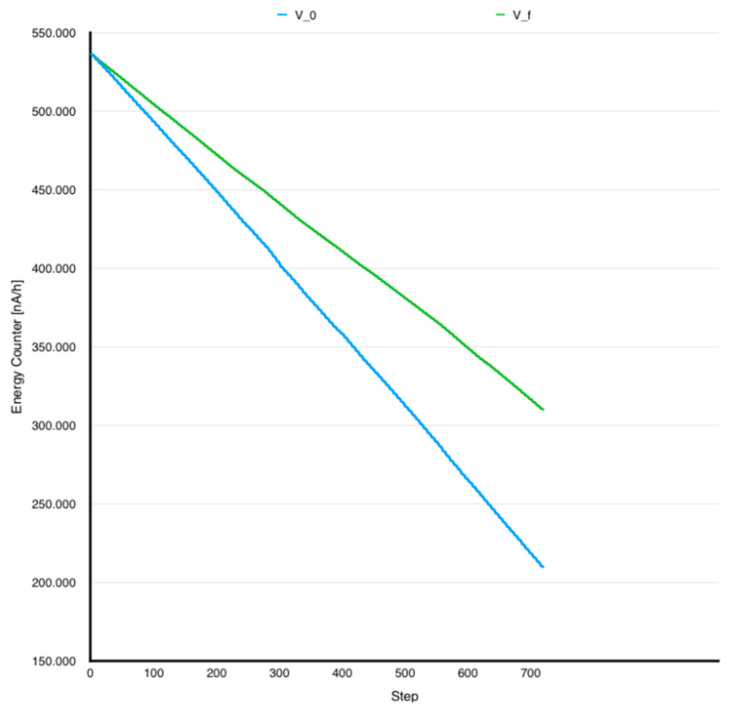
Charge counter unfolding V_0_ vs. V_f_.

Figure 4 presents the charge counter attribute evolution for test executions in two conditions: initial application with all the smells inside and the same application with all the smells solved using the correct solutions. Table 4 shows the percentage values in terms of differences between tested codes.

The following observations were made based on previously analyzed data:

- Corrected versions were more energy efficient, likewise capacity analysis, as shown previously.
- Charge counter traces provided a higher resolution in power consumption measurement and an opportunity to analyze short execution scenarios where capacity remained unchanged.
- Code smells power consumption trends were maintained as capacity analysis suggested.
- Charge counter accuracy enabled a more precise control of the scenario execution starting point.
- Maximum and minimum limit values provided information regarding battery health and physical capacity.

### 5.4. Battery Voltage

**Figure 5:**
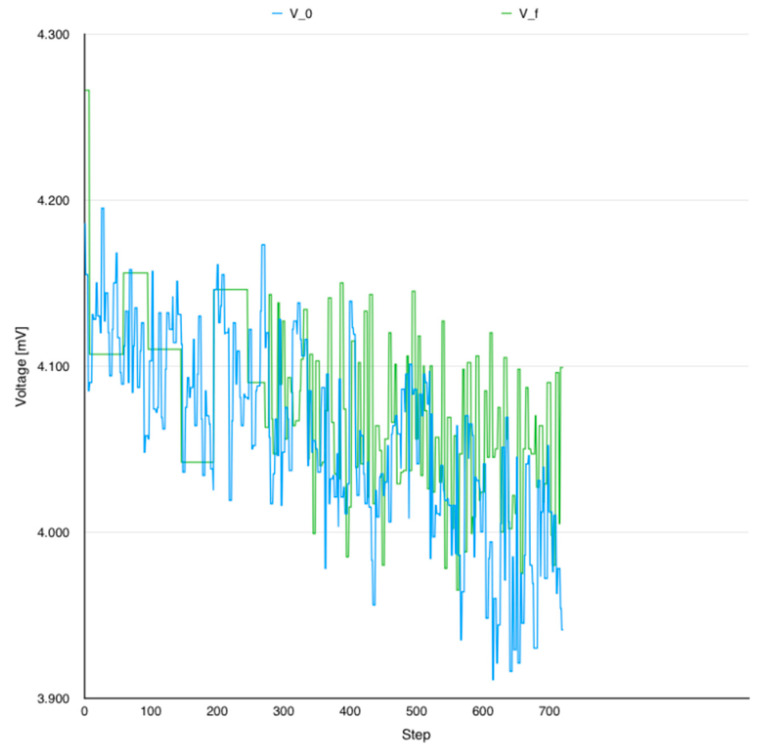
Voltage unfolding V_0_ vs. V_f_.

Figure 5 illustrates battery voltage evolution per application flavor executions in the same extreme conditions: initial application with all the smells inside and the same application with all the smells solved using the correct solutions. Table 5 shows a wide comparison including all the operators defined earlier.

The following observations were made based on previously analyzed data:

- Voltage characteristic was known to be nonlinear. However, analyzed data could give some useful hints on power consumption evolution.
- IGS was the only flavor which showed no improvement with regard to energy efficiency.
- Voltage trace analysis seemed to be more reliable in long execution test scenarios.

### 5.5. Current Consumption

**Figure 6:**
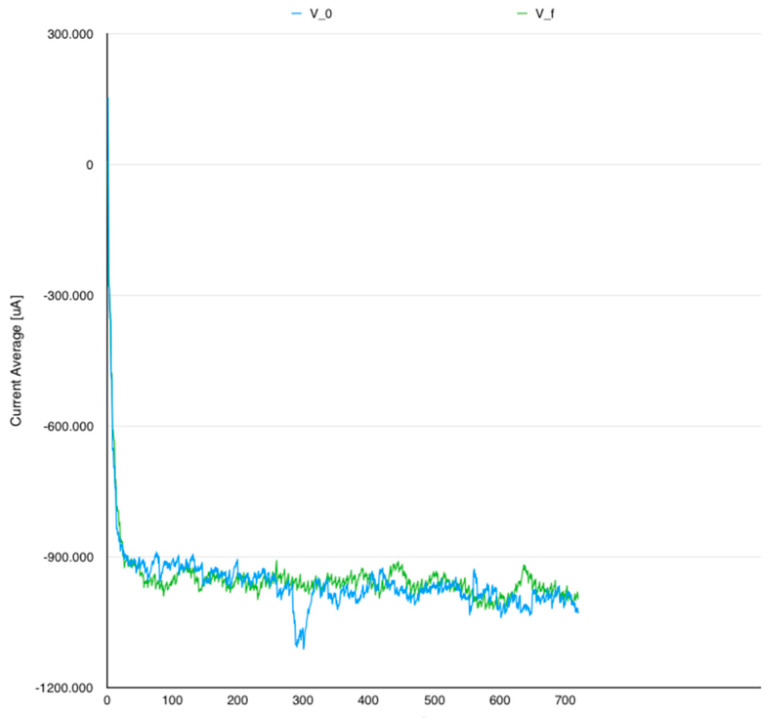
Average current unfolding V_0_ vs. V_f_.

Figure 6 illustrates average current characteristic measurements and Table 6 and Table 7 show a side-by-side comparison in terms of average and median values.

Average current analysis shows little energy efficiency improvement between V_0_ and V_f_. However, the other comparisons are not able to indicate any relevant information in the examination. The following observations were made based on examined dataset:

- Instantaneous current analysis pointed out a slight energy improvement in the instant current characteristic.
- Both the analysis in question and the average current analysis indicated an energy efficiency regression when looking at VHMU flavor. However, when examining the bigger picture, the power consumption in VHMU had been seriously reduced. The first belief for this problem was that VHMU had a better execution time, but it was more resilient in terms of computational power.

## 6. Conclusions

In this paper, a software framework for measuring the energy consumption of mobile applications running on the Android OS is introduced. Three examples have been tested, measured, and analyzed under various conditions and parameters. Energy consumption parameters that can be measured in real-time while running any mobile app are addressed by RQ1. The examples considered for the tests are three code smells commonly found in unoptimized apps, as described by RQ2. In our tests, we demonstrated that resolving code smell issues also reduces the overall impact of the mobile app on battery life. The final outcome of the paper, addressed by RQ3, specifies that energy analysis is an effective indicator for identifying and detecting code smells in sections of code proposed for optimization in a mobile app.

**RQ1: Which are the recommended battery characteristics to be analyzed in order to spot power consumption changes?** All studied battery properties indicated patterns in power consumption tracing. The recommended battery characteristics are classified into short-term and long-term categories:

- **Short term**:
  ○ Charge counter;
  ○ Average current;
  ○ Instantaneous current;
  ○ Energy counter.
- **Long term**:
  ○ Charge counter;
  ○ Capacity;
  ○ Voltage.

It is evident that the charge counter was beneficial for both short-term and long-term analysis. Although the energy counter was an extension of the charge counter and could also be used to assess energy consumption, its use was limited due to the fact that only a few devices support this type of property calculation.

**RQ2: Is there a quantizable correlation between energy code smells and power consumption for mobile applications?** Yes. For answering RQ2, Section 4 manages to demonstrate the existence of power consumption patterns between different code smell app flavors. For example, Table 2 captures some improvements in energy efficiency before and after the correction of one or more energy smells.

**RQ3: Can the software power consumption signature be used to assess the quality of different versions of an application?** Yes. Thus, the software power consumption signature is a set of characteristics that can describe the evolution of power consumption given a specific test scenario inspired by user.

For studying differences between application execution scenarios, a toolset of equations and their application was defined in Section 4. Any combination of resulting data or observed models can generate power consumption signatures for a specific scenario executed on a specific device. Although our experimental results did not reach statistical significance, they show clear trends toward improved energy efficiency through the correction of energy code smells. These findings align with our paper’s objective of equipping developers with actionable insights and tools to reduce power consumption in their applications.

Finally, it can be said that different application versions can generate different power consumption classifications. These patterns can make a major contribution to the quality delivered to an end user.

Reliability of the measurements: The reliability of this work depends on the following

factors:

- The accuracy and limitations of the battery fuel gauging system integrated in a mobile device;
- The invariance of battery properties in respect to close temperature environments;
- Lint static code analysis and manual correction.

## Figures and Tables

**Table 1:** Code version mapping to the type of smell.

Code Version	Fixed Smell
V_0_	-
V_HMU_	HMU
V_IGS_	IGS
V_MIM_	MIM
V_f_	HMU, IGS, MIM

**Table 2:** Battery API properties.

API Constant	Description
BATTERY_PROPERTY_CHARGE_COUNTER	Remaining battery capacity in micro-ampere hour.
BATTERY_PROPERTY_CURRENT_NOW	Instantaneous battery current consumption in microamperes.
BATTERY_PROPERTY_CURRENT_AVERAGE	Average battery current in microamperes.
BATTERY_PROPERTY_CAPACITY	Remaining battery capacity as an integer percentage.
BATTERY_PROPERTY_ENERGY_COUNTER	Remaining energy in nanowatt-hours.
BATTERY_PROPERTY_VOLTAGE	Voltage in millivolts.
BATTERY_PROPERTY_TEMPERATURE	Temperature in degrees Celsius.

**Table 3:** Battery capacity difference reported to V_0_.

Flavor	D_c_ [%]
V_HMU_	−27.27
V_IGS_	−9.09
V_MIM_	−18.18
V_f_	−36.35

**Table 4:** Charge counter difference reported to V_0_.

Flavor	D_cc_ [%]
V_HMU_	−25.25
V_IGS_	−2.94
V_MIM_	−14.94
V_f_	−30.66

**Table 5:** Voltage difference reported to V_0_.

Flavor	Dv [%]
V_HMU_	−51.02
V_IGS_	+8.16
V_MIM_	−4.90
V_f_	−31.84

**Table 6:** Average current.

Flavor	ACavg [mA]	ACmed [mA]
V_0_	959.6	970.5
V_HMU_	974.2	981.1
V_IGS_	952.7	958.5
V_MIM_	981.9	989.6
V_f_	950.7	960.3

**Table 7:** Instantaneous current.

Flavor	ICavg [mA]	ICmed [mA]
V_0_	668.9	641.2
V_HMU_	672.5	653.3
V_IGS_	666.4	646.6
V_MIM_	645.4	629.6
V_f_	657.5	634.9

## Data Availability

Data are contained within the article.
